# Observation of Dispersion in the Japanese Coastal Area of Released ^90^Sr, ^134^Cs, and ^137^Cs from the Fukushima Daiichi Nuclear Power Plant to the Sea in 2013

**DOI:** 10.3390/ijerph16214094

**Published:** 2019-10-24

**Authors:** Hirofumi Tazoe, Takeyasu Yamagata, Kazuki Tsujita, Hisao Nagai, Hajime Obata, Daisuke Tsumune, Jota Kanda, Masatoshi Yamada

**Affiliations:** 1 Department of Radiation Chemistry, Institute of Radiation Emergency Mediation, Hirosaki University, Hirosaki 036-8652, Japan; myamada@hirosaki-u.ac.jp; 2 College of Humanities and Sciences, Nihon University, Tokyo 156-8550, Japan; yamagata@chs.nihon-u.ac.jp (T.Y.); hnagai@chs.nihon-u.ac.jp (H.N.); 3 Graduate School of Basic Integrated Sciences, Nihon University, Tokyo 156-8550, Japan; kaz.tsujita.chs@gmail.com; 4 Atmosphere and Ocean Research Institute, University of Tokyo 277-8564, Japan; obata@aori.u-tokyo.ac.jp; 5 Environmental Science Research Laboratory, Central Research Institute of Electric Power Industry, Tokyo 270-1194, Japan; tsumune@criepi.denken.or.jp; 6 Department of Ocean Sciences, Graduate Faculty of Marine Science, Tokyo University of Marine Science and Technology, Tokyo 108-8477, Japan; jkanda@kaiyodai.ac.jp; 7 Marine Ecology Research Institute, Chiba 299-5105, Japan

**Keywords:** Fukushima Daiichi Nuclear Power Plant, strontium-90, cesium-137, seawater monitoring, contaminated water

## Abstract

The March 2011 earthquake and tsunami resulted in significant damage to the Fukushima Daiichi Nuclear Power Plant (FDNPP) and the subsequent release of radionuclides into the ocean. Here, we investigated the spatial distribution of strontium-90 (^90^Sr) and cesium-134/cesium-137 (^134, 137^Cs) in surface seawater of the coastal region near the FDNPP. In the coastal region, ^90^Sr activity was high, from 0.89 to 29.13 mBq L^−1^, with detectable FDNPP site-derived ^134^Cs. This indicated that release of ^90^Sr from the power plant was ongoing even in May 2013, as was that of ^134^Cs and ^137^Cs. ^90^Sr activities measured at open ocean sites corresponded to background derived from atmospheric nuclear weapons testing fallout. The FDNPP site-derived ^90^Sr/^137^Cs activity ratios in seawater were much higher than those in the direct discharge event in March 2011, in river input, and in seabed sediment; those ratios showed large variability, ranging from 0.16 to 0.64 despite a short sampling period. This FDNPP site-derived ^90^Sr/^137^Cs activity ratio suggests that these radionuclides were mainly derived from stagnant water in the reactor and turbine buildings of the FDNPP, while a different source with a low ^90^Sr/^137^Cs ratio could contribute to and produce the temporal variability of the ^90^Sr/^137^Cs ratio in coastal water. We estimated the release rate of ^90^Sr from the power plant as 9.6 ± 6.1 GBq day^−1^ in May 2013 on the basis of the relationship between ^90^Sr and ^137^Cs activity (^90^Sr/^137^Cs = 0.66 ± 0.05) and ^137^Cs release rate.

## 1. Introduction

Large amounts of radionuclides, such as cesium-134 (^134^Cs), cesium-137 (^137^Cs), and iodine-131 (^131^I), were dispersed into the terrestrial and aquatic environments as a result of an accident at the Fukushima Daiichi Nuclear Power Plant (FDNPP) of the Tokyo Electric Power Company (TEPCO) in March 2011. Atmospheric release of strontium-90 (^90^Sr) in March 2011 was two to four orders of magnitude lower than that of ^137^Cs on the basis of an analysis of highly contaminated soils (<1.1 Bq g^−1^) and vegetation (0.026–1.1 Bq g^−1^) collected from a contaminated area in Japan [1]. These ^90^Sr/^137^Cs activity ratios were much lower than the ratio for the estimated nuclear fuel compositions (^90^Sr/^137^Cs = 0.74) found in the reactor obtained by the ORIGEN2 code [2]. Atmospheric ^90^Sr release (0.01–0.14 PBq [3]) was estimated at less than 0.027% of the total amount in the nuclear fuel (5.2 × 10^2^ PBq [2]) at FDNPP reactor units 1, 2, and 3. Most of the ^90^Sr remained in the reactor, although some of it had dissolved in stagnant water in the reactor and turbine buildings. Observed ^90^Sr and ^137^Cs concentrations in the stagnant water were 140 MBq L^−1^ and 2.8 GBq L^−1^, respectively, on 27 March 2011 [4]. Hence, ^137^Cs concentrations were 20 times higher than ^90^Sr concentrations, and 1.6% of the ^90^Sr core inventory was dissolved into stagnant water [2], which was the most likely candidate for pollution to the ocean. ^90^Sr in seawater could be a useful tracer specific to the radionuclide contaminants directly released from the FDNPP into the ocean.

Analytical results of the stagnant water sampled from a turbine building in February 2012 indicated that ^137^Cs activity decreased to 240 MBq L^−1^, while ^90^Sr concentration remained high (170 MBq L^−1^) [5]. Highly contaminated stagnant water was decontaminated and stored in storage tanks on the FDNPP site. Some decontaminated water was transferred into reactors for cooling purposes after distillation or reverse osmosis processes. Before 2015, the decontamination system was optimized to remove Cs; hence, the treated water had significantly higher ^90^Sr activity (150 MBq L^−1^) than ^137^Cs activity (3.9 kBq L^−1^) [5]. This treated water in the storage tanks was a potential source for ^90^Sr contamination in the environment.

In observation wells between the reactor buildings and the harbor, groundwater was also monitored by TEPCO after a leakage event of contaminated water in December 2012 [4]. In particular, ^90^Sr activity in groundwater in the wells near the seawater intake for reactor units 1 and 2 were significantly higher than the ^137^Cs activity (e.g., ^90^Sr: 5 × 10^6^ Bq L^−1^; ^137^Cs: 2.1 × 10^2^ Bq L^−1^ at the no. 1–2 wells on 5 July 2013 [6]). The ^90^Sr-enriched groundwater might have resulted from leakage of the decontamination system or from stagnant water. Due to these existing contamination sources, it is necessary to observe the ^90^Sr behavior in the aquatic environment near the FDNPP.

**Figure 1:**
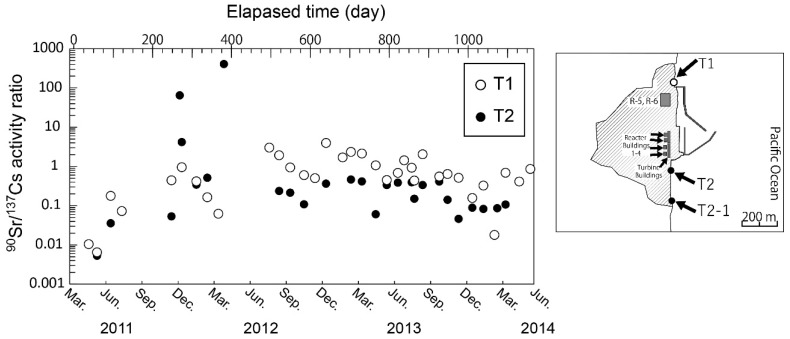
Temporal variations of strontium-90/cesium-137 (^90^Sr/^137^Cs) activity ratio in seawater from monitoring sites T1 and T2 (T2-1) in the Fukushima Daiichi Nuclear Power Plant (FDNPP) site [6]. T1 and T2 (T2-1) sites are located north and south of the discharge channel of the FDNPP, respectively.

Kanda [7] indicated that continuous release of ^137^Cs from the FDNPP harbor to the ocean was occurring in 2012 based on time series seawater monitoring data. Due to the high ^90^Sr/^137^Cs activity ratio in the stagnant water, ^90^Sr release from the FDNPP should also be evaluated. TEPCO has continued seawater monitoring for ^90^Sr, ^134,137^Cs, and other radionuclides near the FDNPP [3,6]. However, only a few ^90^Sr data were obtained within small areas, particularly after 2012 (Figure 1).

This limited monitoring cannot evaluate how much ^90^Sr was released or its impact on the coastal environment and open ocean.

Time series seawater monitoring by TEPCO of ^90^Sr near the FDNPP was infrequent compared to that for radiocesium [3,6]. Povinec et al. [3] showed that the ^90^Sr/^137^Cs ratio in seawater at a monitoring point near FDNPP increased gradually from 0.01 to 1 between April 2011 and February 2012 (Figure 1), which clearly related with decontamination of stagnant water. The transient increase of ^90^Sr in seawater at the T2 site observed in December 2011 could reflect the leakage event from the ^137^Cs decontamination system [4]. After 2012, the ^90^Sr/^137^Cs ratio remained at a constant value around 0.5 at the T2 site with large variability. ^90^Sr/^137^Cs activity ratios in stagnant water have varied depending on the decontamination of ^134, 137^Cs. The agreement between the temporal variation of ^90^Sr/^137^Cs activity ratio and decontamination of the stagnant water supported the idea that the most probable candidate was the continuous release from reactor buildings of the FDNPP.

The behavior of ^137^Cs in seawater and biota after the accident has been well documented [7,8,9,10,11]. High-density sampling of surface seawater to determine radiocesium activity [12] has been carried out. Kumamoto et al. [13] reported detailed vertical distributions of Fukushima-derived radiocesium along the 149 °E meridian in the western North Pacific. However, distributions of ^90^Sr derived from the FDNPP in the sea have been studied to a significantly lesser extent [3,6,14,15,16,17]. Castrillejo et al. [17] suggested that continuous release of ^90^Sr from the FDNPP was occurring in September 2013 based on simultaneous observations of ^90^Sr and ^137^Cs. The estimated release rate of ^90^Sr was 2.3–8.5 GBq day^−1^, which was 2–3 orders of magnitude larger than river inputs.

It is still necessary to investigate the amount of released ^90^Sr, including its subsequent dispersion from the FDNPP site to the ocean. Simultaneous determinations of ^90^Sr and ^137^Cs in seawater are important for monitoring the release of radionuclides from the reactor buildings and contaminated water from the storage tanks. By comparing ^90^Sr behavior with that of ^137^Cs in the ocean, we studied the input source to the sea and the environmental migration processes of both radionuclides, such as fluvial input, desorption from sediment, and atmospheric deposition. Accumulating environmental data and understanding the dispersion to the coastal and open oceans are necessary to respond to any accidental release during decommissioning of the FDNPP—work that will require more than 30 years. Our aim in this study is to determine the distributions of ^90^Sr, ^134^Cs, and ^137^Cs in 2013 and evaluate the continuous release of radionuclides from the FDNPP to the ocean based on the comprehensive analysis of seawater.

## 2. Surface Current System off Fukushima Coast

**Figure 2:**
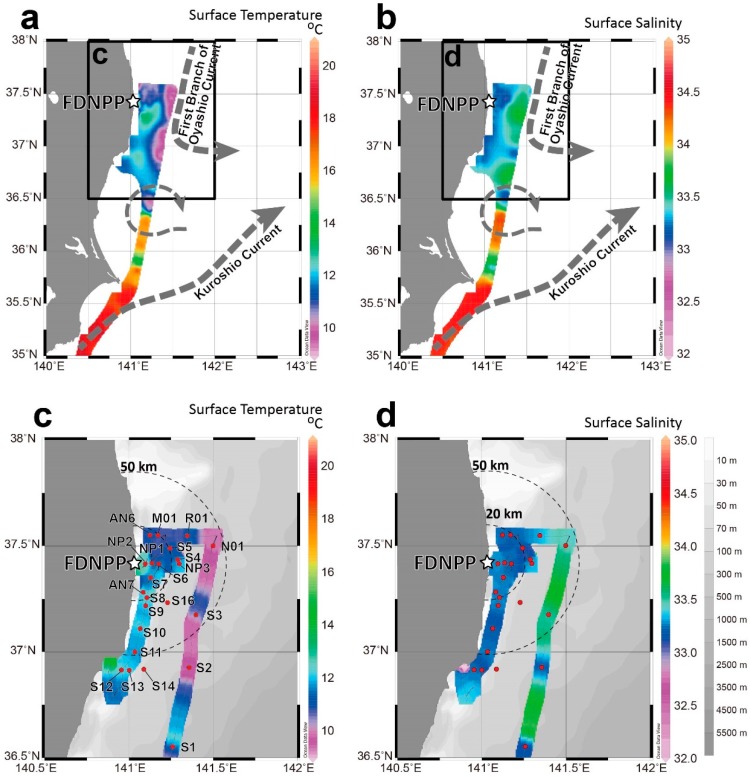
Maps showing sampling locations. (**a**) Surface temperature and (**b**) salinity had two boundary currents (dashed arrows), the warm northeastwardly Kuroshio current south of the Boso Peninsula and the cold southerly first branch of the Oyashio current off the Fukushima coast. (**c**,**d**) Sampling locations are marked by red circles and located near the coast of Fukushima Prefecture.

The Kuroshio and Oyashio currents are generated in the mixed region around 36 °N off the Ibaraki Prefecture coast in the subject area (Figure 2a,b). The warm (16.5–22.0 °C) and saline (34.4–34.8 psu) Kuroshio flows northeastward off the Boso Peninsula. The Oyashio current, off the Fukushima Prefecture coast, intrudes southward into the mixing region. The southward intrusion (9.5–10.5 °C, salinity 33.4–33.8 psu) reaches 36.5 °N, 141.3 °E, and is called the First Branch of the Oyashio [18]. The coastal currents near Fukushima Prefecture are variable on a time scale different from those of the Kuroshio and Oyashio currents. Coastal water is at a higher temperature (10.8–12.7 °C) and lower salinity (33.2–33.4 psu) relative to the first branch of the Oyashio current. Current meter observations made between 1971 and 1981 [19] indicated that the along-shore (north–south component) currents were dominant in this coastal area. The direction of the currents varied approximately every 3−4 days because of changes in the synoptic-scale wind fields [19]. The spread of radionuclides from the direct-release event in April 2011 depended on the coastal current system. Model simulation of directly released Cs employed the Regional Ocean Modeling System (ROMS), which indicated that the plume was southwardly advected to the coastal region [20].

## 3. Materials and Methods

Seawater samples for the analysis of ^90^Sr, ^134^Cs, and ^137^Cs were obtained during the UM13-5 cruise from 14 to 23 May 2013 undertaken by the RTV *Umitaka−Maru* of the Tokyo University of Fisheries, Japan. Seawater sampling sites were located in the offshore region in the first branch of the Oyashio current and the coastal region near Fukushima Prefecture (Figure 2c,d). Most of the coastal sites were south of the FDNPP and close to Iwaki city. The closest observation site to the FDNPP was NP-2, located approximately 6 km east of it. During the sampling period, most of the influence from the FDNPP could be detected in the region associated with the southerly coastal current.

During the cruise, surface seawater samples were collected by an underway sampling system, whose inlet was located on the bottom of the ship, at a depth 5 m below the surface. Collected samples were filtered through a 0.5 μm pore polypropylene cartridge filter (TCW-05N-PPS, Advantec, Tokyo, Japan). Filtered water samples were stored in 20 L polyethylene bags and ^90^Sr and radiocesium analyses were carried out separately on land.

We conducted ^134^Cs and ^137^Cs analyses based on Aoyama et al. [21]. First, 20 L of a filtered seawater sample was acidified to pH 1.6 with HNO_3_. Next, 0.26 g of CsCl was added and the solution was adsorbed on ammonium phosphomolybdate (AMP) [21]. Then, AMP was collected by filtering through a 0.45 μm pore mixed cellulose esters membrane (A045A047A, Advantec, Tokyo, Japan). After drying the AMP/Cs compound, gamma rays were counted for 80,000–200,000 s with a lead-shielded HPGe detector (EGPC 250-P 15, EURISYS MEASURES, NV, USA), 604.7 keV for ^134^Cs and 661.7 keV for ^137^Cs, at the Nihon University in Tokyo. Since the detector was slightly contaminated by atmospherically released ^134^Cs and ^137^Cs at the time of the accident of the FDNPP, the background was determined before and after this counting period and subtracted from the detected signals for seawater samples. Cs yield was determined gravimetrically based on AMP weight. The typical minimum detectable concentrations (MDCs) of ^134^Cs and ^137^Cs were 0.5 mBq L^−1^ and 0.4 mBq L^−1^, respectively.

For ^90^Sr analysis, we added 150 g of (NH_4_)_2_C_2_O_4_ H_2_O to 20 L of filtered seawater and shook the solution vigorously. Sr was precipitated with Ca oxalate. Oxalate precipitate was decomposed to carbonate at 550 °C in a muffle oven. Then, the precipitate was dissolved in HCl and diluted to about 200 mL with Milli-Q water. A small portion of sample solution was used for determination of stable Sr yield by ICP-OES (SPECTROBLUE TI, SPECTRO Analytical Instruments GmbH, Kleve, Germany). After secular equilibrium between ^90^Sr and yttrium-90 (^90^Y) (>2 weeks), ^90^Y with stable Y carrier (0.1 mg) was “milked” from the ^90^Sr by precipitating the Fe hydroxide and purified by solid phase extraction using DGA Resin (DN1ML-R50-S) purchased from Eichrom Technologies, LLC. (IL, USA). Detailed chemical separation and beta counting procedures are described elsewhere [22,23]. Beta particles were counted by a low background 2π gas flow proportional counter (LB−4200, Canberra, NV, USA) during 120 min intervals for more than 20 h. Typical Sr and Y yields were 82 ± 9 % and 95 ± 5 %, respectively.

## 4. Results

Activities of ^90^Sr, ^134^Cs, and ^137^Cs in surface seawater samples collected in May 2013 are summarized in Table 1. Mean ^90^Sr activity of 0.80 ± 0.11 mBq L^−1^ at offshore sites (S1, S2, S3, and N01) was slightly lower than the estimated value (1.0 ± 0.1 mBq L^−1^ [3]) based on long-term monitoring for surface water of the western North Pacific. Around sites S2, S3, and N01, cool surface water (9.4–10.4 °C) from the southerly first branch of the Oyashio current was present. ^134^Cs activities were lower than the MDC (<0.5 mBq L^−1^) at the offshore sites. In this study, we used values obtained at the offshore sites as the background level originating from atmospheric nuclear weapons testing. Compared to ^137^Cs and ^90^Sr, ^134^Cs has a relatively short half-life (2.06 years compared to 30.17 years for ^137^Cs and 28.8 years for ^90^Sr).

High ^90^Sr activities were observed along the coastal region with higher temperatures (10.86–12.89 °C) and higher salinity (33.23–33.35 psu). The highest ^90^Sr activity (29.13 ± 0.35 mBq L^−1^) was found at AN7, approximately 16 km south of the FDNPP, with 22.4 ± 0.6 mBq L^−1^ for ^134^Cs activity and 44.7 ± 0.4 mBq L^−1^ for ^137^Cs activity. At the NP-2 site closest to the FDNPP (5 km offshore) in this sampling campaign, we also found high ^90^Sr activity (21.81 ± 0.28 mBq L^−1^). Furthermore, at S12 off Iwaki City, 57 km south of the FDNPP, relatively high ^90^Sr activity (9.86 ± 0.22 mBq L^−1^) was found. Distributions of radiocesium activities in surface seawater showed similar trends to those of ^90^Sr. The maximum radiocesium activities were obtained at AN7; in particular, ^137^Cs activities ranged from 1.4 mBq L^−1^ at S2 to 44.7 mBq L^−1^ there. In the coastal region, ^134^Cs activities were in agreement with ^137^Cs activities corrected to 11 March 2011, which indicated that this radiocesium was derived from the Fukushima accident (^134^Cs/^137^Cs = 0.99 ± 0.03 [11]).

## 5. Discussion

### 5.1. Dispersion of High ^90^Sr and ^134, 137^Cs Activity Plume

**Figure 3:**
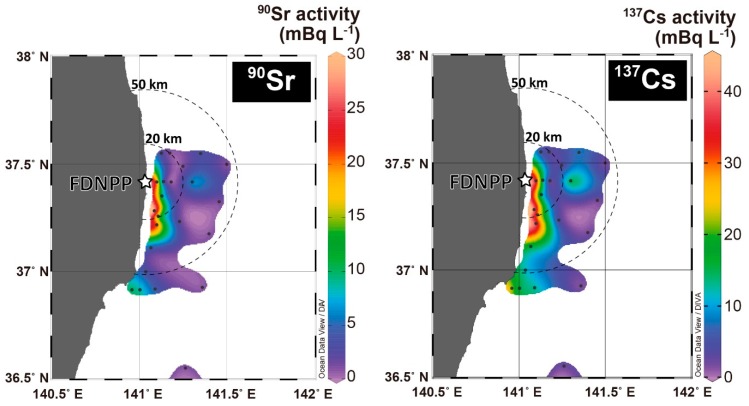
Distribution of ^90^Sr and ^137^Cs activities in surface seawater collected in May 2013.

The high activities of ^90^Sr and ^137^Cs in the coastal region (Figure 3) can be explained by the release at the time of the FDNPP accident and the physical processes that later occurred in the ocean. In the coastal region, high ^90^Sr activity seawater samples with high ^134^Cs and ^137^Cs activities were mainly collected from south of the FDNPP (NP2, and AN7) to off Iwaki (S12 and S6), which reflects the southward transport of seawater along the Fukushima coast by the coastal currents. Higher ^90^Sr activity in seawater (>8 mBq L^−1^) was found where the salinity was 33.23–33.33 psu and 11.95–12.41 psu. In the coastal region, no clear correlation between ^90^Sr and salinity or ^90^Sr and temperature was observed.

The distributions of ^90^Sr and ^137^Cs activities in May 2013 observed in this study correspond to those of a model simulation of the direct-release event between 26 March and 6 April 2011 [20]. The ^137^Cs released from the FDNPP from 26 March was initially advected southward, then transported to the Ibaraki coast. This simulation suggested that the ^137^Cs concentration decreased in May due to advection and diffusion in the open ocean. The coastal currents are variable in this region and sometimes flow northward. Oikawa et al. [16] compiled monitoring data for seawater in the coastal region by the Ministry of Education, Culture, Sports, Science and Technology, Japan (MEXT) and suggested that the activities of ^90^Sr in surface water decreased slowly over time in 2011 and reached the background level by the end of December 2011. However, because of a lack of sampling sites for ^90^Sr, the ^90^Sr plume could have been missed in previous observations. The distribution of our results indicates that the released ^90^Sr plume from the FDNPP site (leakage of contaminated water from storage tanks) could move to the coastal region south of the FDNPP, carried by the southward coastal current.

Both activities decreased rapidly from NP2 toward the eastern sites (S6, NP1, and NP3), which indicate that the eastward dispersion was limited because of the effect of the southern coastal current during this sampling period. Compared with the pre-Fukushima accident, offshore ^90^Sr activities in the north Pacific Ocean (1.0 ± 0.1) [3] and the activities measured in May 2013 indicate that the influences of the Fukushima-derived ^90^Sr on open ocean sites in the mixed region between the Oyashio and Kuroshio currents were negligibly small, as were those of ^134^Cs and ^137^Cs. However, if any accidental releases from the FDNPP site were to occur during the decommissioning of the reactors, coastal areas could be exposed to a high activity plume.

In September 2017, low ^90^Sr (1.0−1.8 mBq L^−1^) and high ^137^Cs (9−43 mBq L^−1^) activities were obtained at low salinity (4–28 psu) in groundwater and beach seawater samples from Sendai Bay, located north of the FDNPP [17]. ^90^Sr/^137^Cs ratios ranged from 0.036 to 0.19. The ^137^Cs activity in low salinity samples was affected by atmospheric fallout from the FDNPP accident that was deposited on land, while ^90^Sr activity was not sensitive to terrestrial input. Therefore, the relationship between ^90^Sr and ^137^Cs can be a useful indicator for river input.

The mouth of the Ukedo River is located between collection sites NP2 and AN6. ^137^Cs activity at NP2 (39.0 mBq L^−1^) was more than five times higher than that at AN6 (6.9 mBq L^−1^), though their salinities were comparable (33.24 and 33.26 psu). Dissolved ^137^Cs activity in the Ukedo River, which drains a highly contaminated area, ranged from 200 to 1100 Bq L^−1^ in August and November 2012 [6]. ^90^Sr activity in the Ukedo River was not available but the reported ^90^Sr/^137^Cs ratio for river water in the Fukushima Prefecture [6] was less than 0.04. The contribution to ^90^Sr activity in seawater by input from the Ukedo River should be minor.

### 5.2. ^90^Sr/^137^Cs Activity Ratios Derived from the FDNPP Accident

**Figure 4:**
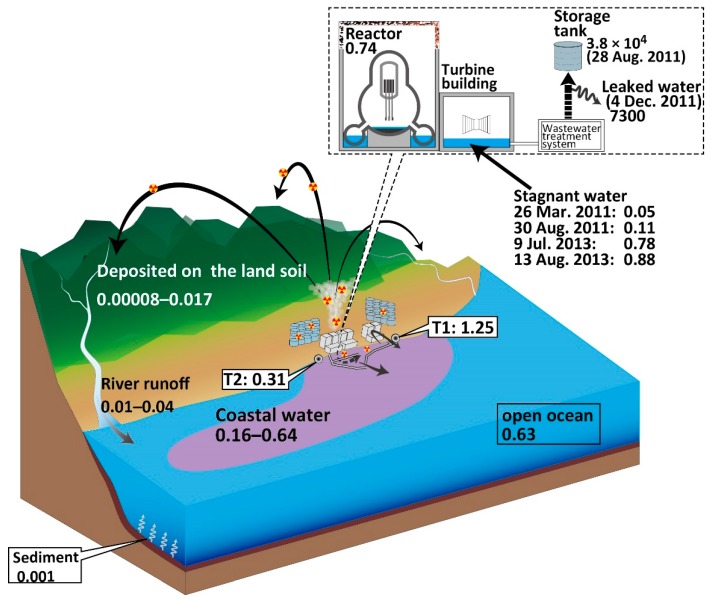
The ^90^Sr/^137^Cs activity ratios in the environment and the FDNPP site. The ^90^Sr/^137^Cs activity ratio in seawater near the FDNPP was consistent with those from the monitoring points, T1 and T2 [12]. Soil [4] and sediment [24] samples had extremely low ^90^Sr/^137^Cs activity ratios.

The ^90^Sr/^137^Cs activity ratios in seawater are different according to timing of any release or leakage event (e.g., direct discharge event from late March to early April 2011 [3,15]). Since Sr and Cs are highly soluble in seawater, the ^90^Sr/^137^Cs activity ratio depends on the source, which could be a useful tracer for the source. The most possible source of ^90^Sr and ^137^Cs is stagnant water in the reactor building of unit 2. The ^90^Sr/^137^Cs ratio of open ocean seawater [3], seawater monitoring data near the FDNPP [6], stagnant water [4], atmospheric input [1], seabed sediment [24], and river water [6] are summarized with our data in Figure 4.

The ^90^Sr activity of 0.80 ± 0.11 mBq L^−1^ was obtained at the offshore sites, S1, S2, S3, and N01 (Table 2). To evaluate FDNPP site-derived ^90^Sr, measured ^90^Sr activity was subtracted from this value as the background value for North Pacific seawater. The measured ^134^Cs was a pure FDNPP site-derived component because of its short half-life (*T*_1/2_ = 2.06 yr). The FDNPP site-derived ^134^Cs/^137^Cs ratio was reported to be 0.99 ± 0.03 [11] in March 2011. FDNPP site-derived ^137^Cs was calculated on the basis of measured ^134^Cs activity and the FDNPP site-derived ^134^Cs/^137^Cs ratio.

**Figure 5:**
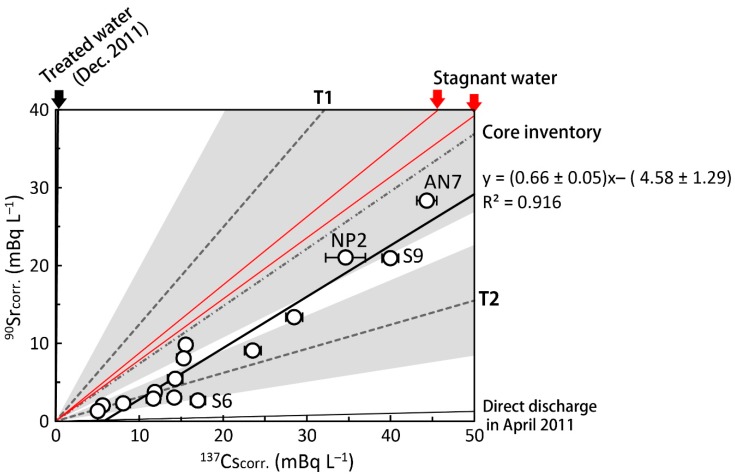
FDNPP site-derived ^90^Sr and ^137^Cs activities in surface seawater with ^90^Sr/^137^Cs ratio for possible sources. The lower and upper red solid lines show ^90^Sr/^137^Cs ratios for stagnant water in the reactor building in July 2013 (0.78) and August 2013 (0.88), respectively [6]. Core inventory (0.74) was estimated by the ORIGEN2 code [2]. Shaded areas were averaged monitoring data at T1 (1.25 ± 0.71) and T2-1 (0.31 ± 0.14) sites from January 2013 to October 2013.

The ^90^Sr_corr_/^137^Cs_corr_ ratio estimated from the slope of a linear regression fitting was 0.66 ± 0.05 in Figure 5. ^90^Sr_corr_ activities strongly correlated with those of ^137^Cs_corr_ (R^2^ = 0.919), as described in similar contour maps of ^90^Sr and ^137^Cs (Figure 3). The high correlation between ^90^Sr and ^137^Cs indicates that ^90^Sr and ^137^Cs were derived from a common source. However, the low ^90^Sr activity samples (<10 mBq L^−1^) showed larger variability in ^90^Sr/^137^Cs activity ratio (0.34 ± 0.14) relative to those for high ^90^Sr activity samples (>10 mBq L^−1^: ratio of 0.56 ± 0.08). If multiple sources to seawater exist, such as stagnant water, storage water, and groundwater, contributions from each source could yield temporal and spatial variations. To distinguish these components, detailed ^134,137^Cs and ^90^Sr distributions should be investigated. Castrillejo et al. [17] found a short-term transition of ^90^Sr/^137^Cs ratio from 0.14 to 0.36 and an abrupt increase in ^137^Cs activity in the vicinity of the FDNPP (observation site St. 1 (or NP0)) in September 2013. ^90^Sr and ^137^Cs release from the FDNPP could be related to the tidal cycle and weather conditions, which caused a temporal variation of the released ^90^Sr/^137^Cs ratio from the FDNPP site.

The slope of a linear regression fitting (0.66) was similar to the reported ^90^Sr/^137^Cs activity ratios in stagnant water of 0.78 and 0.88, respectively, in July and August of 2013 [5]. The stagnant water samples were collected from the sampling line behind the mixing point of water from each reactor building [4,5] (Figure 5). These radionuclides were thought to mainly be derived from the reactor building of unit 2 on the basis of the initial data for stagnant water in the unit 2 turbine building [2], which was severely damaged. The ^90^Sr/^137^Cs activity ratio in stagnant water varied depending on the decontamination of ^134, 137^Cs, and gradually increased from the direct-release event in March 2011 (0.0256 ± 0.0006 [15]). The ^90^Sr/^137^Cs activity ratio of seawater in this study is slightly lower than that of stagnant water, although the most possible source candidate is the continuous release of stagnant water from the FDNPP.

The discrepancy between our data and monitoring data at T1 (1.25 ± 0.71) implies multiple sources exist at the FDNPP site. The higher ^90^Sr/^137^Cs at the T1 site could reflect a contribution from ^90^Sr-rich groundwater. Groundwater around the reactor buildings had a ^90^Sr/^137^Cs activity ratio (2.4 × 10^4^ [5]) that was 5 orders of magnitude higher than the seawater value observed in this study. The decontaminated water in storage tanks in the FDNPP was also observed to have high ^90^Sr/^137^Cs activity ratios.

The fitted regression line had an x-intercept of 5.8 mBq L^−1^. A very low ^90^Sr/^137^Cs ratio (0.16) was observed at S6 without a change in salinity. These results indicate that there is a missing source for the site with a low ^90^Sr/^137^Cs ratio. In the coastal region, salinities ranged from 33.2 to 33.3 psu and showed no correlation with activities of ^90^Sr and ^134, 137^Cs. Atmospherically deposited ^90^Sr on land soil in March 2011 was at a lower level (<1.1 Bq g^−1^) than ^137^Cs, where the ^90^Sr/^137^Cs activity ratio was considered to be 0.00008−0.017 [1] (Figure 5). Higher mobility of ^90^Sr has been recognized, but ^90^Sr activity in water of the Fukushima River was less than 4 mBq L^−1^ in 2012 [6]. ^137^Cs activity ranged from 12 to 190 mBq L^−1^, which yielded a low ^90^Sr/^137^Cs activity ratio of 0.01–0.04 [6]. Considering the ^90^Sr/^137^Cs activity ratio in seawater, riverine input from the land to the ocean was minor for ^90^Sr, though dissolved ^90^Sr activity in the Ukedo River was never reported.

A possible supply process for ^137^Cs is the release from seabed sediments. Some amount of Cs could be scavenged by seabed sediments through adsorption onto particles, such as clay minerals [25,26,27] during the direct discharge event. The sedimentary ^137^Cs inventory of 100–200 TBq represents only 1%–3% of the total discharge from the FDNPP to the Pacific Ocean in 2011 [28,29]. Approximately 80% of the total ^137^Cs sedimentary inventory was found in coastal sediments at less than 150 m water depth [29]. The highest ^90^Sr activity of 63 Bq kg-dry^−1^ in seabed sediments was observed near the south discharge gate of the FDNPP site (T2 monitoring point) in September 2011 [6]. Sedimentary ^90^Sr/^137^Cs activity ratios observed after the accident ranged from 0.001 to 0.08, which were lower than those in seawater. ^137^Cs could be attributed to the direct discharge event in late March to April 2011 [20]. The extremely low ^90^Sr/^137^Cs ratio indicates that the contribution of ^90^Sr in seawater from the soil and seafloor sediments is less than that of ^137^Cs, even if there is a higher mobility for Sr than for Cs in the soil and sediments.

Another possible low ^90^Sr/^137^Cs source is contaminated water that remained in a tunnel for pipes and cables, which were connected to the turbine buildings of units 2 and 3. Contaminated water in the turbine and reactor buildings was released into the ocean via the tunnel and cracks resulting from the earthquake and tsunami. During this direct release event, the ^90^Sr/^137^Cs ratio was very low 0.0256 ± 0.0006 [15] (Figure 5). ^137^Cs activity at T1 reached 68 kBq L^−1^ [20]. After the direct release was stopped in early April 2011 by sealing cracks and the tunnel entrance, contaminated water could have been left in the tunnel until July 2015. Such highly contaminated water could be the source of the low ^90^Sr/^137^Cs ratio.

The ^90^Sr/^137^Cs activity ratio of 0.66 ± 0.05 observed in this study was higher than data at the monitoring point, T2-1, near the south discharge gate (0.31 ± 0.14) [6] from January to December 2013, but was lower than that at T1 near the north discharge gate (1.25 ± 0.71) [6] (Figure 5). A large variation of ^90^Sr/^137^Cs at T1 was observed, which might reflect the local input processes of ^90^Sr and ^137^Cs. A much higher ^90^Sr/^137^Cs activity ratio (e.g., ^90^Sr activity of 7.5 Bq L^−1^ and ^90^Sr/^137^Cs activity ratio of 3.2 on 26 June 2013) was observed by TEPCO in the harbor [6] than the coastal region as observed in this study. The variation in ^90^Sr/^137^Cs activity ratios might reflect the spatial and temporal heterogeneities of released water.

As mentioned above, the similarity of the ^90^Sr/^137^Cs activity ratio between seawater and the stagnant water supported the idea that the most likely candidate was the continuous release from the reactor buildings of the FDNPP. Both high ^90^Sr activity and ^90^Sr/^137^Cs activity ratio in the coastal region reflect the input of the stagnant water. Variability of ^90^Sr/^137^Cs activity ratios in seawater is an important indicator to understand the status of the release of contaminated water from the FDNPP. Unfortunately, the contribution of underground water near the reactor buildings, and released from sediments to the harbor water, could not be distinguished from the release of the reactor buildings on the basis of seawater obtained from outside of the harbor. More detailed temporal and spatial data in the harbor and for other radionuclides such as tritium (^3^H) and iodine-129 (^129^I) are necessary.

### 5.3. Estimation of ^90^Sr Input to the Ocean from the FDNPP

The continuous release from the FDNPP was the main source to the Fukushima coast. The amount of ^137^Cs released daily to the ocean was estimated to be from 8.1 GBq day^−1^ [7] to 30 GBq day^−1^ [8] in 2012 on the basis of simulation of the ^137^Cs activities of seawater in the harbor and at the north discharge gate, respectively. We examined the amount of released ^90^Sr based on that of ^137^Cs in 2013 by using Equation (1):

(1) $$N_{Sr-90}=N_{Cs-137}\times {\left(\frac{C_{Sr-90}}{C_{Cs-137}}\right)}_{SW}=C_{Cs-137}\times F\times {\left(\frac{C_{Sr-90}}{C_{Cs-137}}\right)}_{SW}$$

where *N*, *C*, and *F* represent release rate, activity, and conversion factor from activity to daily release rate of ^137^Cs, respectively. For the estimation of daily released ^137^Cs, the activities of ^137^Cs at T2-1 300 m south of the south discharge gate were used (Table 3) [6]. Most of the ^137^Cs activities were lower than the MDA (1.2–1.5 Bq L^−1^). To avoid overestimation of the averaged ^137^Cs activity, we used only precise analysis data. The ^137^Cs activity at the T2-1 site ranged from 0.14 to 0.98 Bq L^−1^ and showed considerable variation (mean value = 0.60 ± 0.35 Bq L^−1^). The conversion factors, *F*, from activity to daily release rate of ^137^Cs were obtained on the basis of the amount of released ^137^Cs in the direct release event of March 2011 and ^137^Cs activities at the T2-1 site [7,20]. The conversion factor applied was 25.5 × 10^9^ [20] for the T2-1 site.

The resulting daily released amount of ^90^Sr was 9.1 ± 6.1 GBq day^−1^ during our sampling campaign in May 2013. The observed ^90^Sr activity in the coastal region was too low to disturb the ecological system and affect the background radiation dose, as mentioned above. Continuous release could increase the inventory of ^90^Sr in the Pacific Ocean. If the constant release (9.1 GBq day^−1^) continued over the year, the annual release rate would be estimated at 3.3 TBq yr^−1^, which is small relative to the inventory of 105 PBq in the ocean [3]. However, ^90^Sr in seawater should be closely observed to detect any unexpected release from the nuclear reactor buildings and the contaminated water storage tanks. This estimation needs to assume a stable release rate from the single source. As discussed above, the low ^90^Sr/^137^Cs source contributed to seawater around the FDNPP. Therefore, this result could be overestimated.

In this study, the ^90^Sr/^137^Cs activity ratio of 0.66 ± 0.05, which was influenced by continuous release from the FDNPP, was distinguished based on precise ^90^Sr analysis. Buesseler et al. [11,30] suggested that the possible source of ^137^Cs was not only continuous release from the FDNPP but also the input from subsurface groundwater [31], river water [32], and desorption from the marine sediments in the coastal region [29,30,33]. The environmental migration of ^137^Cs through particulate and dissolved fluvial inputs, and remineralization from the sediments contaminated by direct discharge of stagnant water from 26 March to 6 April 2011, must also be taken into consideration. ^90^Sr/^137^Cs activity ratios could fluctuate according to the source in the FDNPP area and remobilization of ^137^Cs in coastal water. In addition to monitoring for ongoing release from the reactor buildings and possible leakage of stored contaminated water in tanks, continuous measurement of ^90^Sr is necessary for investigation of the migration of ^137^Cs in the marine environment. A combination of other fission product nuclides, ^129^I and ^3^H activity, will provide precise information for the current status of leakages from stagnant water, groundwater, and stored water in tanks.

### 5.4. Estimation of Effective Dose Rate by Ingestion from Marine Products

^90^Sr dispersion to the coastal area is the most serious issue for fisheries due to its radiotoxicity. We estimated the dose impact to human health from marine products. The highest ^90^Sr activity (29.13 mBq L^−1^ at AN7; Table 1) was comparable to typical levels for North Pacific surface seawater in the early 1960s during nuclear weapons testing [34]. Taking into consideration the processes in the food chain and the highest activity in the coastal water observed in this study (29.13 mBq L^−1^ at AN−7), we obtained Equation (2):

(2) D=C×CF×IR×F

where *D* is representative of the annual dose rate. *C*, *CF*, *IR*, and *F* are representative of the ^90^Sr activity in seawater, the concentration factor from seawater to marine products (5–10 [35]), the intake rate of marine products (28.4 kg yr^−1^ [36]), and dose coefficient (2.8 × 10^−8^ Sv/Bq [37]), respectively. It should be noted that these concentrations are quite small (0.23 μSv yr^−1^) compared with the International Commission on Radiological Protection (ICRP) limit of 1 mSv yr^−1^ for a member of the general public. Much higher ^90^Sr activities were observed at monitoring points near the south (150−670 mBq L^−1^) and north (260−5800 mBq L^−1^) discharge gates [6]. Even this anomalously high ^90^Sr activity (5800 mBq L^−1^) close to the FDNPP would contribute 46 μSv yr^−1^ to the annual effective dose rate by marine products.

## 6. Conclusions

^90^Sr is useful as a tracer for continuous releases from the FDNPP site. We reported ^90^Sr data in seawater along with ^134^Cs and ^137^Cs in samples collected in the coastal area off Fukushima Prefecture. Released ^90^Sr was dispersed along the Fukushima coast, and the highest ^90^Sr activity was 29.13 mBq L^−1^ at a sampling site 16 km south of the FDNPP. FDNPP site-derived ^90^Sr/^137^Cs ranged from 0.16 to 0.64 and the slope of a linear regression fitting of the relationship of Fukushima site-derived ^90^Sr and ^137^Cs was 0.66 ± 0.05, which was similar to the ratio of contaminated water in the FDNPP reactor and turbine buildings. These results suggest that the major contamination source is contaminated water in the FDNPP buildings. On the other hand, the ^l37^Cs-rich source could also affect seawater and cause temporal and spatial variations. The estimated release rate of ^90^Sr (9.6 ± 6.1 GBq day^−1^) was small relative to the inventory of ^90^Sr in the Pacific Ocean. Release of ^90^Sr has been controlled by the water shielding wall between the reactor buildings and the harbor since 2015. However, our results imply that if any accidental release of radionuclides, including ^90^Sr from the FDNPP, occurs during decommissioning of the reactors, the coastal area can be exposed to a high activity plume.

## Figures and Tables

**Table 1:** ^90^Sr, ^134^Cd, and ^137^Cs activities and hydrographic data in seawater collected on the Fukushima Prefecture coast in May 2013. Uncertainties represent 1σ error.

ID	Sampling Date and Time (year/month/day)	Latitude	Latitude	Temperature (^o^C)	Salinity (psu)	^90^Sr Activity (mBq L^−1^)	^134^Cs Activity (mBq L^−1^)	^137^Cs Activity (mBq L^−1^)
S1	2013/5/14 16:41	36°33.15′ N	141°15.60′ E	11.41	33.16	0.87 ± 0.06	<0.4	1.9 ± 0.1
S2	2013/5/14 18:39	36°55.62′ N	141°21.48′ E	9.43	33.21	0.66 ± 0.05	<0.4	1.4 ± 0.1
S3	2013/5/14 19:54	37°10.50′ N	141°23.88′ E	10.37	33.51	0.75 ± 0.06	<0.4	1.8 ± 0.1
S4	2013/5/14 20:47	37°26.13′ N	141°17.46′ E	9.88	33.64	1.03 ± 0.06	<0.4	1.5 ± 0.1
N01	2013/5/14 23:12	37°29.33′ N	141°14.75′ E	9.72	33.46	0.90 ± 0.07	<0.4	2.0 ± 0.2
R01	2013/5/15 5:39	37°25.20′ N	141° 8.26′ E	11.02	33.31	3.09 ± 0.07	4.1 ± 0.4	7.7 ± 0.3
AN6	2013/5/15 7:53	37°21.09′ N	141° 7.80′ E	10.86	33.24	2.94 ± 0.13	6.9 ± 0.4	6.9 ± 0.2
M01	2013/5/16 2:28	37°15.42′ N	141° 6.40′ E	10.37	33.24	0.92 ± 0.07	1.0 ± 0.3	3.8 ± 0.2
NP3	2013/5/16 8:05	37°13.11′ N	141° 5.94′ E	11.90	33.35	6.25 ± 0.08	7.2 ± 0.5	14.5 ± 0.3
S5	2013/5/16 6:36	37° 6.60′ N	141° 4.02′ E	11.22	33.17	0.89 ± 0.06	1.4 ± 0.3	3.5 ± 0.2
S6	2013/5/16 6:08	36°59.97′ N	141° 2.16′ E	11.09	33.26	3.46 ± 0.08	8.6 ± 0.5	18.2 ± 0.3
NP2	2013/5/16 22:05	36°54.93′ N	140°57.36′ E	12.41	33.26	21.81 ± 0.28	17.5 ± 1.2	39.0 ± 1.2
NP1	2013/5/17 3:48	36°54.87′ N	141° 0.06′ E	11.08	33.21	0.99 ± 0.07	1.4 ± 0.2	3.8 ± 0.1
S7	2013/5/17 11:27	36°55.05′ N	141° 5.35′ E	11.98	33.28	14.17 ± 0.23	7.9 ± 0.4	16.5 ± 0.3
S8	2013/5/17 12:08	37°16.94′ N	141° 5.14′ E	12.10	33.26	10.63 ± 0.27	14.4 ± 0.5	29.8 ± 0.4
S9	2013/5/17 12:40	37°14.00′ N	141°13.80′ E	11.95	33.24	21.74 ± 0.38	20.2 ± 0.5	39.8 ± 0.4
S10	2013/5/17 14:05	37°32.91′ N	141°20.85′ E	12.04	33.21	3.84 ± 0.12	7.2 ± 0.4	15.7 ± 0.3
S11	2013/5/17 15:11	37°33.03′ N	141°10.41′ E	11.74	33.24	3.68 ± 0.08	5.9 ± 0.4	12.5 ± 0.3
S12	2013/5/17 16:15	37°24.99′ N	141°17.99′ E	12.36	33.26	9.86 ± 0.22	11.9 ± 0.5	24.0 ± 0.3
S13	2013/5/19 0:57	37°33.03′ N	141° 7.56′ E	12.32	33.23	8.92 ± 0.25	7.7 ± 0.4	16.1 ± 0.3
S14	2013/5/19 5:09	37°24.99′ N	141°10.68′ E	12.69	33.30	4.56 ± 0.08	6.0 ± 0.4	13.3 ± 0.3
AN7	2013/5/20 8:45	37°24.99′ N	141° 5.88′ E	12.20	33.33	29.13 ± 0.35	22.4 ± 0.6	44.7 ± 0.4
S16	2013/5/20 23:05	37°30.00′ N	141°30.00′ E	12.16	33.26	2.10 ± 0.06	2.6 ± 0.3	5.7 ± 0.2

**Table 2:** FDNPP site-derived ^90^Sr (^90^Sr_corr._) and ^137^Cs (^137^Cs_corr._) activities and ^90^Sr_corr._/^137^Cs_corr._ activity ratios.

ID	^90^Sr_corr._ Activity (mBq L^−1^)	^137^Cs_corr._ Activity (mBq L^−1^)	^90^Sr_corr._/^137^Cs_corr._
S6	2.66 ± 0.13	17.0 ± 1.0	0.16 ± 0.01
S7	9.83 ± 0.28	15.6 ± 0.8	0.63 ± 0.04
S8	13.37 ± 0.25	28.5 ± 1.0	0.47 ± 0.02
S9	20.94 ± 0.39	40.0 ± 1.0	0.52 ± 0.02
S10	3.04 ± 0.16	14.2 ± 0.8	0.21 ± 0.02
S11	2.88 ± 0.13	11.7 ± 0.8	0.25 ± 0.02
S12	9.07 ± 0.24	23.5 ± 1.0	0.39 ± 0.02
S13	8.12 ± 0.27	15.2 ± 0.8	0.53 ± 0.03
S14	3.76 ± 0.13	11.9 ± 0.8	0.32 ± 0.02
AN7	28.33 ± 0.37	44.3 ± 1.2	0.64 ± 0.02
S16	1.30 ± 0.12	5.1 ± 0.6	0.25 ± 0.04
R01	2.30 ± 0.12	8.1 ± 0.8	0.28 ± 0.03
NP3	5.46 ± 0.13	14.2 ± 1.0	0.38 ± 0.03
AN6	2.15 ± 0.16	5.9 ± 0.6	0.36 ± 0.05
NP2	21.02 ± 0.30	34.6 ± 2.4	0.61 ± 0.04

**Table 3:** Estimation of the release rates for ^90^Sr and ^137^Cs into the ocean from the FDNPP site in May 2013. *C*: activity; *F*: conversion factor; *N*: release rate.

Monitoring Point	$C_{Cs-137}\;(\mathrm{Bq}\;L^{-1})$ (Jan. to Oct. 2013)	F (×10^9^ L day^−1^)	$N_{Cs-137}$ (GBq day^−1^)	${\left(\frac{C_{Sr-90}}{C_{Cs-137}}\right)}_{SW}$	$N_{Sr-90}$ $\left(\mathrm{GBq}\;{\mathrm{day}}^{-1}\right)$
T2-1	0.60 ± 0.35 [6]	25.5 [20]	15.3 ± 8.9	0.63 ± 0.05	9.6 ± 6.1
